# New approaches to secondary metabolite discovery from anaerobic gut microbes

**DOI:** 10.1007/s00253-024-13393-y

**Published:** 2025-01-20

**Authors:** Lazarina V. Butkovich, Oliver B. Vining, Michelle A. O’Malley

**Affiliations:** 1 Department of Chemical Engineering, University of California, Santa Barbara, CA 93106 USA; 2 Institute for Collaborative Biotechnologies, University of California, Santa Barbara, CA 93106 USA; 3 U.S. Department of Energy Joint Genome Institute (JGI), Lawrence Berkeley National Laboratory, Berkeley, CA 94720 USA

**Keywords:** Gut microbiome, Secondary metabolite, Biofoundry, Genome mining, Anaerobic

## Abstract

**Abstract:**

The animal gut microbiome is a complex system of diverse, predominantly anaerobic microbiota with secondary metabolite potential. These metabolites likely play roles in shaping microbial community membership and influencing animal host health. As such, novel secondary metabolites from gut microbes hold significant biotechnological and therapeutic interest. Despite their potential, gut microbes are largely untapped for secondary metabolites, with gut fungi and obligate anaerobes being particularly under-explored. To advance understanding of these metabolites, culture-based and (meta)genome-based approaches are essential. Culture-based approaches enable isolation, cultivation, and direct study of gut microbes, and (meta)genome-based approaches utilize *in* *silico* tools to mine biosynthetic gene clusters (BGCs) from microbes that have not yet been successfully cultured. In this mini-review, we highlight recent innovations in this area, including anaerobic biofoundries like ExFAB, the NSF BioFoundry for Extreme & Exceptional Fungi, Archaea, and Bacteria. These facilities enable high-throughput workflows to study oxygen-sensitive microbes and biosynthetic machinery. Such recent advances promise to improve our understanding of the gut microbiome and its secondary metabolism.

**Key points:**

*• Gut microbial secondary metabolites have therapeutic and biotechnological potential*

*• Culture- and (meta)genome-based workflows drive gut anaerobe metabolite discovery*

*• Anaerobic biofoundries enable high-throughput workflows for metabolite discovery*

**Graphical abstract:**

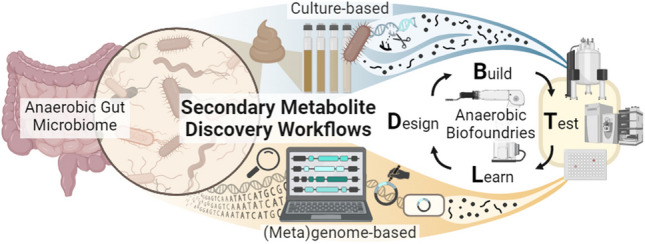

## Introduction

The animal gut microbiome is an evolutionary marvel, and its complexity and anaerobic nature pose a challenge to its understanding. The scale of bioinformatic wealth in gut microbiota is staggering: in an individual human for example, the gut microbiota consists of trillions of cells spanning over 500 species, harboring a gene pool that surpasses the human genome by over 100-fold (Sender et al. 2016; Gilbert et al. 2018). The vast majority of gut microbiota consist of viruses and bacteria, although lower abundance members such as fungi, archaea, and protozoa also play key roles. Over millions of years of co-evolution, host animals and gut microbiota have achieved symbiosis, with gut microbiota driving outcomes in host health, including protection from pathogens, immune system regulation, and digestion, with many of these roles mediated by a landscape of microbially produced secondary metabolites (Yang and Cong 2021; Cheng et al. 2024).

**Fig. 1:**
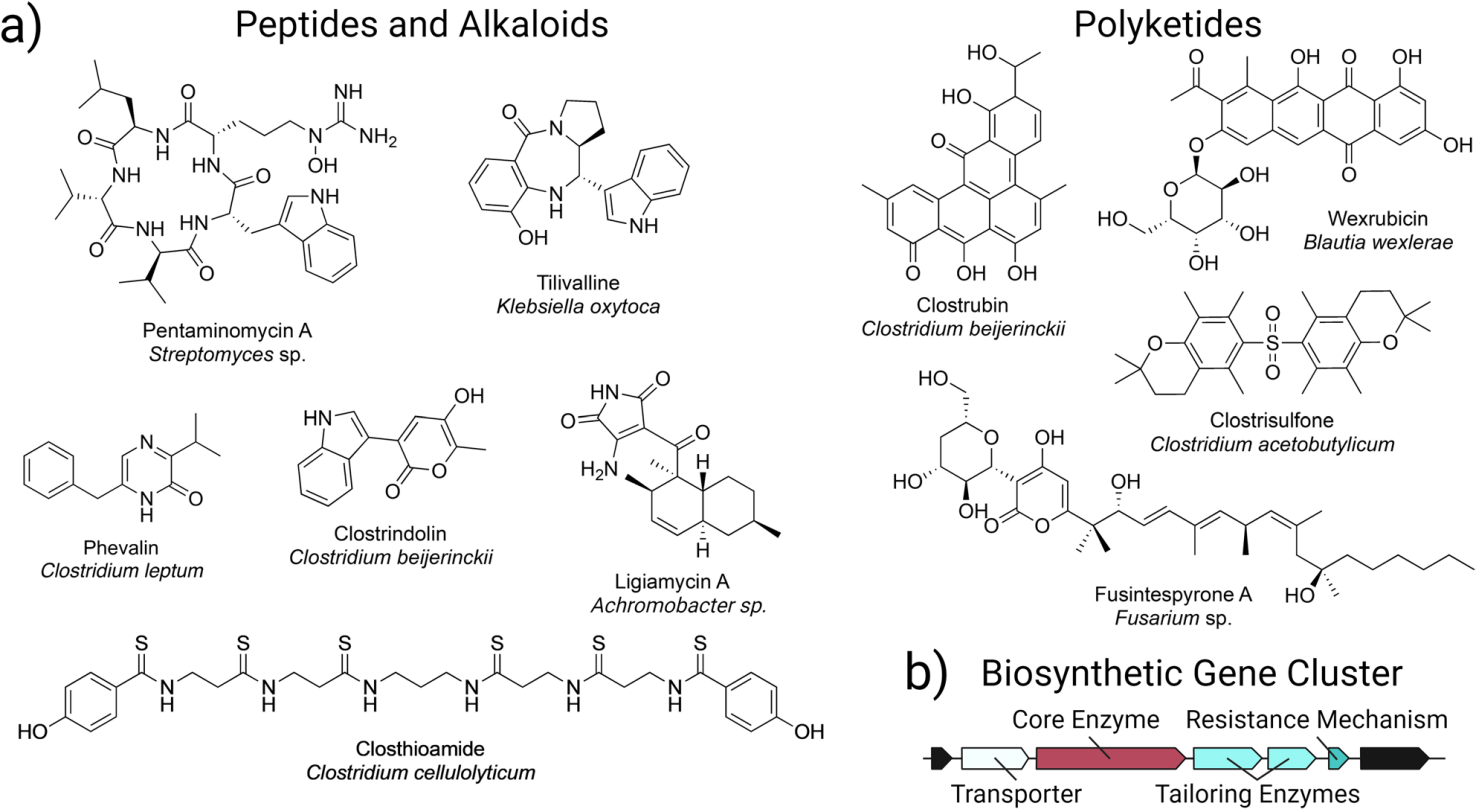
Recent studies highlight anaerobic microbes as a source for novel secondary metabolites.** a** Pentaminomycin A, tilivalline, phevalin, ligiamycin, wexrubicin, and fusintespyrone A were characterized from gut microbes, and clostrindolin, closthioamide, clostrubin, and clostrisulfone were characterized from obligately anaerobic *Clostridium* from soil. **b** Biosynthetic gene clusters (BGCs) encode the machinery to produce secondary metabolites. BGCs consist of co-localized genes that include (i) at least one core biosynthetic gene encoding a biosynthetic enzyme and (ii) accessory genes for gene regulation, tailoring enzymes, transporters, and self-resistance mechanisms. Figure was created with BioRender.com

Secondary metabolites, also frequently referred to as natural products or specialized metabolites, are structurally diverse, organic small-molecules (< 3000 Da) that can possess bioactivities, such as antibiotic, anti-cancer, immunosuppressant, and anti-viral activity (Craney et al. 2013). Secondary metabolites play a variety of native roles, including pigmentation (Narsing Rao et al. 2017), defense (Isah 2019), virulence factors (Vogt et al. 2015), quorum signaling (Rangel and Bolton 2022), and microbe-microbe and microbe-host interactions (Cruz et al. 2022). As a result of their unique chemistries, secondary metabolites have applications in pesticides, preservatives, biopolymers, drop-in biofuels, and pharmaceuticals, with over 60% of today’s pharmaceuticals related to secondary metabolites (Keswani et al. 2020; Newman and Cragg 2020; Kim et al. 2021b; Mosquera et al. 2021; Keasling et al. 2021). Secondary metabolites are categorized into several classes, including polyketides, non-ribosomal peptides, ribosomally synthesized and post-translationally modified peptides (RiPPs), alkaloids, and terpenes, and the biosynthetic pathways to produce secondary metabolites are typically encoded by biosynthetic gene clusters (BGCs) (Fig. 1).

Secondary metabolites from gut microbiota likely drive important outcomes in host health and function, and they further could be tapped for their biotech potential as anti-inflammatories, antimicrobials, and postbiotics, products from probiotic microbes. Additionally, microbiome complexity correlates with diversity and abundance of secondary metabolites, which reflect increased metabolic exchange and microbial interactions in processes like quorum sensing, symbiosis, and chemical warfare (Phelan et al. 2012). Understanding the complex gut secondary metabolome is relevant for human health, as secondary metabolites can strongly modulate microbial community membership (Hatziioanou et al. 2017; Chevrette et al. 2022; Duncan et al. 2023). Gut dysbiosis has been linked to a range of human diseases (Winter and Bäumler 2023): cardiovascular disease (Karlsson et al. 2012), diabetes (Wang et al. 2012; Ceccarani et al. 2020), colorectal cancer (Scott et al. 2019), and inflammatory bowel disease (Frank et al. 2007). In silico studies and experimental validations further support the understanding that the complex gut microbiome possesses untapped secondary metabolite potential (Wang et al. 2019; Garcia-Gutierrez et al. 2019).

Given the interest surrounding secondary metabolites from gut microbiota, recent advances offer promising directions for studying and scaling production of these metabolites. Lab automation is increasingly pursued to accelerate scientific discoveries, and biofoundries—automated facilities focused on enabling synthetic biology—are a central example. Biofoundries are not typically designed for fully anaerobic workflows, but two recent facilities specialize in anaerobic studies: the NSF BioFoundry for Extreme & Exceptional Fungi, Archaea and Bacteria (ExFAB) (exfab.org) and the LanzaTech biofoundry (lanzatech.com). Regarding current bioproduction, industrial-scale aerobic bioreactors fully or semi-synthesize important secondary metabolite-based therapeutics, including artemisinin (Ro et al. 2006; Paddon and Keasling 2014) and lovastatin (Bizukojc and Ledakowicz 2008; Mulder et al. 2015). Anaerobic bioreactors improve upon aerobic designs, due to significant reductions in energy requirements for mixing, aeration, and heat removal (Weusthuis et al. 2011; Cueto-Rojas et al. 2015; Humbird et al. 2017). Additionally, anaerobic, acetogenic bacteria such as *Clostridium* are promising industrial workhorses for sustainable secondary metabolite production, using CO and H_2_/CO_2_ gases as a carbon source (Zhang et al. 2024a). Overall, discovering biosynthetic machinery for valuable secondary metabolites from anaerobes has the potential to make industrial processes more sustainable.

**Fig. 2:**
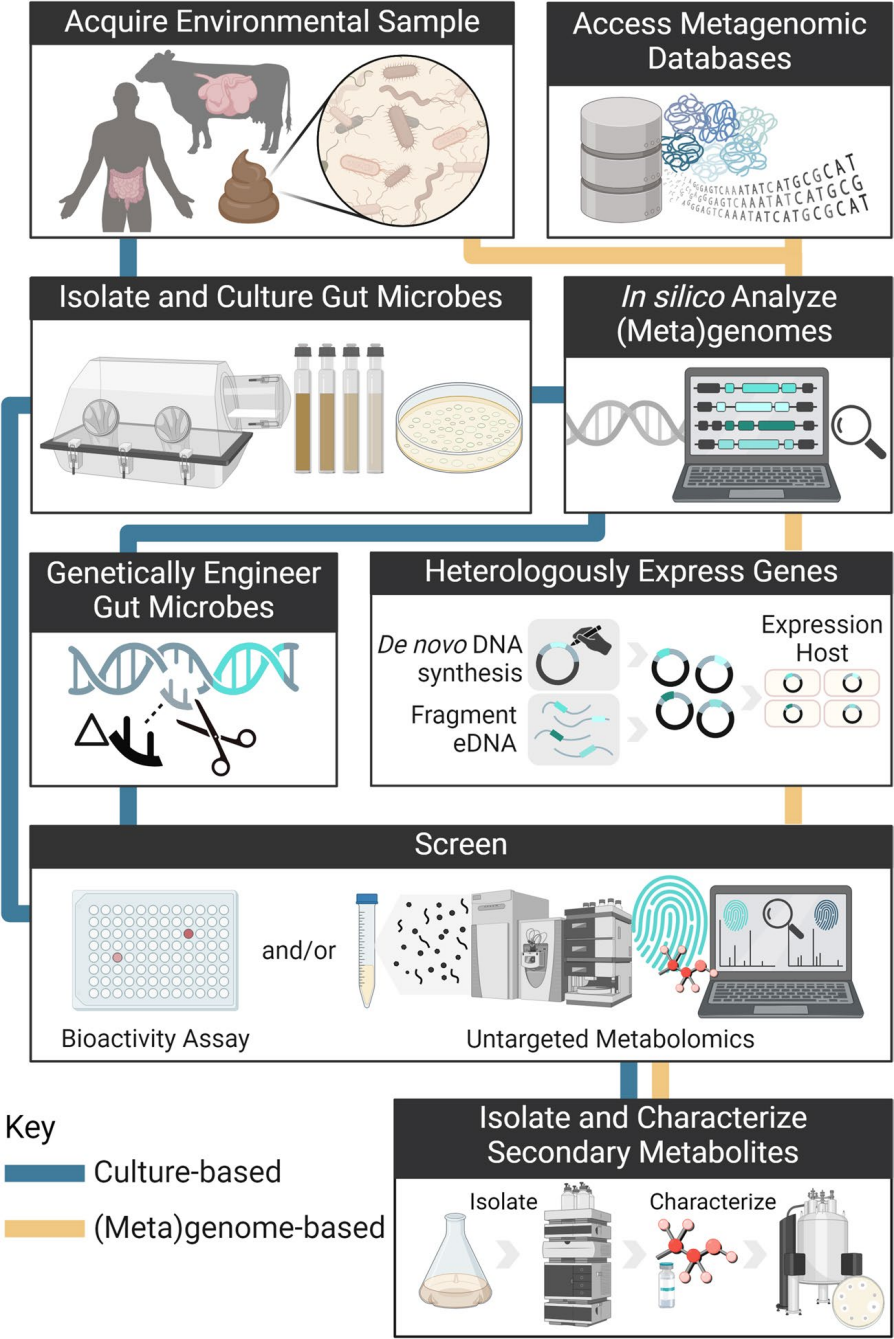
Culture-based and (meta)genome-based workflows drive exploration for novel secondary metabolites from nature. (i) The culture-based method screens successfully cultured gut microbes, with the option to genetically engineer them. (ii) The (meta)genome-based workflow mines gut microbial genomic sequences for predicted secondary metabolite BGCs, using sequences from environmental samples, cultured microbes, or public databases. Heterologous expression techniques enable the study of mined BGCs in well-studied expression hosts or cell-free systems. Figure was created with BioRender.com

In this mini-review, we provide an overview of current approaches for secondary metabolite mining from gut microbes, spotlighting obligate anaerobes, gut fungi, and opportunities to accelerate secondary metabolite characterization via anaerobic biofoundries. For a summary of secondary metabolites discovered from mammalian gut bacteria, we refer the reader to other comprehensive reviews (Donia and Fischbach 2015; Wang et al. 2019; Garcia-Gutierrez et al. 2019). In the following sections, we describe culture-based and (meta)genome-based workflows (Fig. 2) that leverage overlapping techniques to screen, isolate, and characterize secondary metabolites from gut microbes.

### Culture-based workflows enable focused secondary metabolite discovery from isolated gut microbes

Successful isolation and cultivation of a microbe significantly expands the techniques available to characterize and enhance production of secondary metabolites from the microbe. Techniques for culture-based workflows (Fig. 2) have been well-described (Xu et al. 2024). Researchers typically isolate gut microbial strains from fresh feces, using serial culturing with (i) agar plate-based techniques (valid only for colony-forming species) or (ii) liquid dilution-based techniques utilizing microfluidics (Du et al. 2009; Overmann 2013; Watterson et al. 2020; Jian et al. 2023). To target specific microbe types, selection pressures can be applied. In rumen microbial enrichments for example, chloramphenicol selects for fungi, penicillin–streptomycin selects for fungi and methanogens, and the combined addition of hydrogen and lack of carbon source selects for prokaryotes (Gilmore et al. 2019). After isolation, robust cryopreservation and cryo-revival methods are essential to maintaining strains for future studies.

**Fig. 3:**
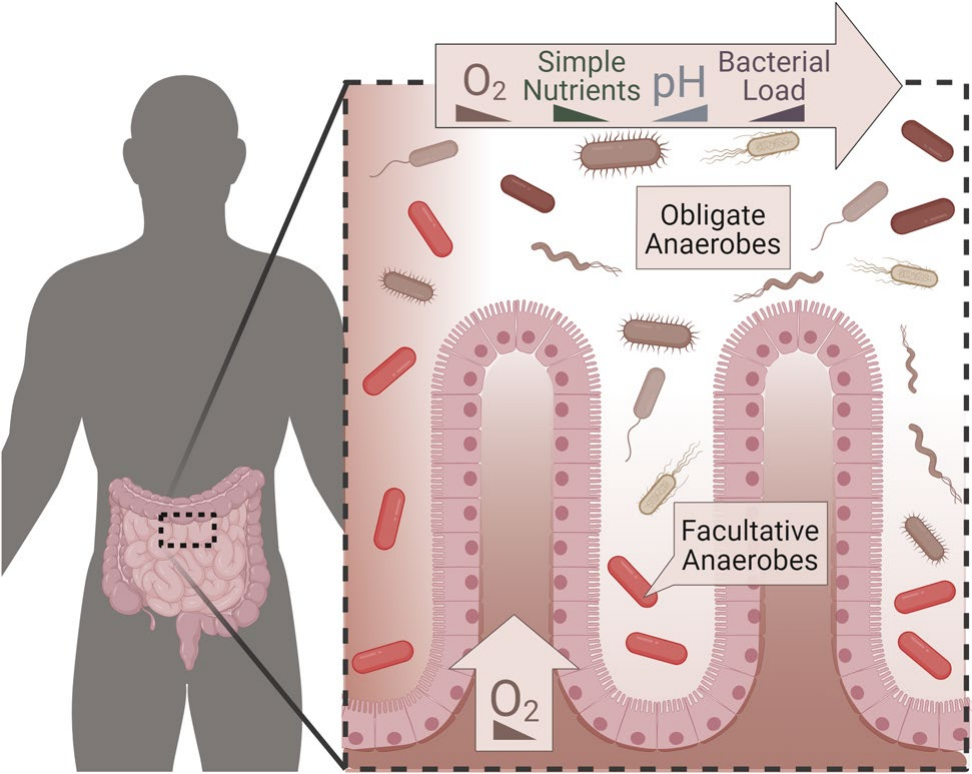
The animal gut possesses steep gradients of oxygen and hosts microbes with ranges of oxygen tolerance. Gut biogeography and oxygen variation have been well-described in animal guts (McCallum and Tropini 2024; Maritan et al. 2024). Oxygen enters the gut via the vasculature at the gut epithelium and via partially digested food contents from the stomach, and oxygen leaves via both facultative anaerobe respiration and host mechanisms, as evidenced by the anoxic guts of germ-free animals (Sonnenburg et al. 2005; Espey 2013; Friedman et al. 2018; Lu and Imlay 2021). Figure was created with BioRender.com

The choice of aerobic or anaerobic culturing strongly affects what microbe types are selected during enrichment and ultimate isolations. The gut is mostly anoxic and hosts a range of obligate and facultative anaerobes and lowly abundant aerobes (Fig. 3). Unlike facultative anaerobes, which survive under ambient oxygen, obligate anaerobes—constituting the majority of human lumen microbiota (von Martels et al. 2017)—generally cannot survive prolonged oxygen exposure, though some can exploit low levels of oxygen (Lu and Imlay 2021). Since facultative anaerobes can survive aerobic culturing, they have been studied more extensively than obligate anaerobes. Anaerobic culturing is relatively more complicated than aerobic culturing, requiring specialized procedures and equipment, such as an anaerobic chamber (Wagner et al. 2019). Temporary, economical options for anaerobic culturing also exist, such as AnaeroPak (Delaney and Onderdonk 1997) and BBL GasPak (Collee et al. 1972), although the ability of these systems to maintain a fully anaerobic state is limited. As an added difficulty, most high-throughput screening methods do not translate well to anaerobes. Characterization by flow cytometry is not possible due to ambient oxygen in unmodified instrument setups (Thompson et al. 2015) and the tendency for anaerobes to form biofilms (Donelli et al. 2012). Additionally, characterization by MALDI-TOF is currently limited by the lack of reference spectra for gut anaerobes (Plomp et al. 2024). Historically, the additional effort, costs, and difficulties are barriers-to-entry for labs to study gut microbes anaerobically and specifically target obligate anaerobes.

Once gut microbes are isolated and cultured, their ability to produce secondary metabolites can be studied directly. Typically, biological assays are performed in parallel with LCMS or NMR-based untargeted/targeted metabolomics to characterize the chemistry and bioactivity of metabolites present in microbial cultures, often across isolate libraries as an initial screen to prioritize further interrogation of specific microbes or sources (Rinschen et al. 2019; Vitale et al. 2024). For example, screens have highlighted gut microbiota from animals in polluted environments as promising sources of novel antibiotics (Akbar et al. 2020; Siddiqui et al. 2023). Additionally, Adra et al. screened 37 termite gut-associated *Streptomyces* isolates for antifungal activity against the plant pathogen *Pyrrhoderma noxium*, and MS/MS-based networking yielded putative identification of some known polyketides and potential novel metabolites (Adra et al. 2023). A major challenge in culture-based workflows is that microbes often do not express BGCs under standard lab conditions, as secondary metabolites are energetically costly to biosynthesize and may require specific biotic or abiotic environmental cues (Covington et al. 2021). Certain methods have proven useful in activating silent BGCs, including “One Strain Many Compounds” (OSMAC) techniques (Bode et al. 2002; Pan et al. 2019) and co-culturing (Selegato and Castro-Gamboa 2023). For example, after performing OSMAC on *Streptomyces* sp. GG23 from the guts of the mealworm beetle (*Tenebrio molitor*), Hwang et al. characterized pentaminomycins (Fig. 1), which possessed autophagy-inducing activity and in vitro activity against oxidative stress (Hwang et al. 2020). Additionally, Lim et al. co-cultivated bacterial strains *Streptomyces* sp. GET02.ST and *Achromobacter* sp. GET02.AC from the wharf roach gut (*Ligia exotica*), leading to the discovery of two novel GET02.ST secondary metabolites: the antibacterial ligiamycin A and anticancer ligiamycin B (Fig. 1) (Lim et al. 2022).

Genomic sequencing of cultured gut microbes enables genome-based mining for secondary metabolites (see next section) and direct genetic engineering techniques to establish biosynthetic pathways, engineer promoters, improve access to precursor molecules, and manipulate BGCs (Alam et al. 2021). However, relative to aerobes, tools and techniques to genetically engineer anaerobes are lacking and pose a challenge to develop. Recent studies highlight their potential in secondary metabolite research, such as the use of Tn5 transposon-based mutagenesis by Dornisch et al. to elucidate the biosynthetic pathway of tilivalline (Fig. 1), a toxin from gut bacterium *Klebsiella oxytoca* implicated in human colitis (Dornisch et al. 2017). The creation of novel genetic engineering toolboxes for anaerobes is essential for accelerating the study of gut anaerobes. Ameruoso et al. established a novel approach combining CRISPR interference (CRISPRi) and activation (CRISPRa) to perturb the endogenous regulatory network of the aerobic soil bacterium *Streptomyces* and activate silent BGCs. This genetic engineering toolbox can also be utilized for *Streptomyces* strains present in the gut (Bolourian and Mojtahedi 2018; Ameruoso et al. 2022). In recent years, genetic and metabolic engineering tools in anaerobes have been utilized to improve production of valuable primary metabolites, such as butyrate in commensal gut bacteria (Gong et al. 2023) and hexanol and butanol from syngas (CO_2_/H_2_) in *Clostridium ljungdahlii* (Liew et al. 2016; Hoff et al. 2021; Lauer et al. 2022). Such genetic engineering tools and concepts can also be applied in anaerobes to further interrogate biosynthetic pathways and improve production of secondary metabolites.

### (Meta)genome-based workflows enable secondary metabolite discovery in uncultured gut microbes

While there are clear benefits to working with cultured gut microbial isolates for secondary metabolite discovery, gut microbes are challenging to isolate and culture, with ~ 70% of identified human gut bacteria not yet cultured (Almeida et al. 2021). Failure of gut microbes to grow under standard laboratory conditions is often attributed to a lack of specific factors (nutrients, signaling compounds, physical interactions, etc.), which may be produced by the animal host or native gut microbiome (Epstein 2013). To face this challenge, (meta)genome-based methods (Fig. 2) including in silico genome mining and heterologous expression have been essential in establishing our current understanding of gut microbial metabolism, phylogenies, and untapped secondary metabolite potential.

In silico genome mining tools predict secondary metabolite BGCs, aiding in prioritization of genomic targets for experimental study. Current tools for BGC prediction include antiSMASH 7.0 (Blin et al. 2023), PRISM 4 (Skinnider et al. 2020), TaxiBGC (Gupta et al. 2022), RiPPMiner-Genome (Agrawal et al. 2021), NaPDoS (Klau et al. 2022), and ARTS 2.0 (Mungan et al. 2020). These and similar tools have been well-covered in recent reviews (Li 2023; Wang et al. 2024) and typically function by searching input genomes for conserved sequences of (i) core biosynthetic genes that encode core enzymes or (ii) self-resistance genes that detoxify the secondary metabolite for the producing organism. Sequence-based predictive tools may fail to detect BGCs with insufficient similarity to known sequences, an issue of particular relevance for anaerobes, since most studied BGCs are from aerobic organisms (Letzel et al. 2013, 2014). Alongside tools for secondary metabolite mining, the tool gutSMASH predicts specialized, primary metabolic gene clusters in gut microbial genomes, which can complement studies for secondary metabolite discovery (Pascal Andreu et al. 2023).

In silico genome mining tools can be applied to metagenome-assembled genomes, genomes from cultured microbes, and genomes from public repositories, where large amounts of curated data are now publicly available and pose an accessible resource to prioritize future discovery efforts. Such (meta)genomic repositories for gut microbes include the Human Microbiome Project (HMP) (Huttenhower et al. 2012; Methé et al. 2012) and its second phase, the Integrative Human Microbiome Project (HMP2) (Proctor et al. 2019); the Hungate1000 collection for rumen microbes (Seshadri et al. 2018); the Unified Human Gastrointestinal Genome (UHGG) Collection (Almeida et al. 2021) and its expanded catalog, the Human Reference Gut Microbiome (HRGM) (Kim et al. 2021a); the Animal Microbiome Database (AMDB) (Yang et al. 2022); Metagenomics of Human Intestinal Tract (MetaHIT) (Yang et al. 2022); and the Exposome-Explorer (Neveu et al. 2023). Recent studies report in silico analyses from these and other repositories. For example, Ma et al. recently implemented machine learning to mine antimicrobial peptides from 15 human gut microbiome metagenomic cohorts, prioritizing antimicrobial peptides with low toxicity to human cells and effectiveness against both *Klebsiella pneumoniae* in a lung infection mouse model and multi-drug resistant, gram-negative bacteria (Ma et al. 2022). Similarly, the MetaBGC algorithm was developed by Sugimoto et al. to enable metagenome-based BGC mining. Application of MetaBGC analysis to metagenomes derived from the human gut microbiome resulted in identification of multiple novel secondary metabolites, including wexrubicin (Fig. 1), a novel anthracycline type II polyketide from a gut *Clostridia*, *Blautia wexlerae* DSM 19850 (Sugimoto et al. 2019). Broader efforts to mine genome repositories have yielded BGC databases, such as Minimum Information about a Biosynthetic Gene Cluster (MIBiG) (Terlouw et al. 2023), BiG-FAM (Kautsar et al. 2021), and sBGC-hm (Zou et al. 2023), which organize current information on BGCs.

Heterologous expression is a key technique to explore the in silico-predicted metabolic potential of an organism and involves expressing target genes in a well-characterized host. Target genes can originate from fragmented environmental DNA, cultured microbe genomic DNA, or *de* *novo* synthesized DNA. Although de novo DNA synthesis enables codon-optimization and access to virtually any sequenced gene for heterologous expression, this option is often cost-prohibitive due to the typical large sizes of BGCs. Traditional expression hosts, like *E. coli*, *Streptomyces*, and yeast, can struggle to express BGCs from anaerobes, likely due to differences in regulatory or biosynthetic elements between the source and host microbes (Galm and Shen 2006; Zhang et al. 2017). To address this issue, genetically tractable, anaerobic bacterial host systems have been developed. Hao et al. developed an expression system in the facultative anaerobe *Streptococcus mutans* and utilized the system to genome-mine 10,038 *Streptococcus* strains (Hao et al. 2019). Additionally, Sanford et al. used chassis-independent recombinase-assisted genome engineering (CRAGE) to develop an expression system in *Eubacterium limosum*, an acetogenic *Clostridia* and obligate anaerobe from the human gut. Sanford et al. heterologously expressed a non-ribosomal peptide BGC from the human gut bacteria *Clostridium leptum* to produce phevalin (Fig. 1) (Sanford et al. 2024). Cell-free expression systems are another option to explore in silico-predicted metabolic potential. In 2020, Krüger et al. developed the first cell-free expression system for an obligate anaerobe, *Clostridium autoethanogenum* (Krüger et al. 2020). Overall, these recent developments in anaerobic expression systems will facilitate further secondary metabolite discovery from gut anaerobes.

Both culture-based and (meta)genome-based workflows culminate in efforts to screen, isolate, and structurally elucidate secondary metabolites. For a thorough discussion of the latest techniques involved in these steps, we refer the reader to other resources. Generally, gut microbial and heterologous expression cultures can be screened for secondary metabolites using traditional bioactivity assays (i.e., disc dilution or microdilution assays) and untargeted metabolomics (ie: LC–MS/MS) (Rinschen et al. 2019; Vitale et al. 2024). A major milestone in these discovery efforts is the isolation (i.e., bioactivity-guided fractionation with HPLC) and structural characterization (i.e., NMR, microED) of a novel secondary metabolite (Sarker et al. 2006; Zhao and Yue 2023; Gaudêncio et al. 2023).

### Obligate gut anaerobes and gut fungi are promising sources of secondary metabolites

Obligate anaerobes were long believed to not produce secondary metabolites (Lincke et al. 2010). However, this understanding changed with the discovery of secondary metabolites from *Clostridium*, a bacterial genus found in the soil and gut and utilized in industrial solvent production. In 2010, Lincke et al. discovered closthioamide (Fig. 1), the first nonribosomal peptide discovered from an obligate anaerobe, the soil bacterium *Clostridium cellulolyticum* (Lincke et al. 2010). Shortly after, Pidot et al. discovered clostrubin (Fig. 1), the first polyketide reported from an obligate anaerobe (*Clostridium beijerinckii*), which features a benzo[a]tetraphene ring structure unprecedented in natural product chemistry and exhibited potent antibiotic activity against methicillin-resistant *Staphylococcus aureus* (MRSA), vancomycin-resistant enterococci (VRE), and mycobacteria (Pidot et al. 2014). More recently, the antimycobacterial alkaloid clostrindolin was also discovered from *C. beijerinckii* (Schieferdecker et al. 2019), and clostrisulfone, the first reported natural product with a diphenylsulfone scaffold, was discovered from *C. acetobutylicum* (Fig. 1) (Neuwirth et al. 2020).

While *Clostridium* has been the subject of increasing interest for secondary metabolite discovery, other obligate anaerobes remain largely unexplored. To broadly characterize secondary metabolite potential of obligate anaerobes, Letzel et al. in silico genome-mined 211 bacterial obligate anaerobes, of which 40% were from the *Firmicutes* phylum, which includes *Clostridium*. They found that BGC abundance for polyketides and non-ribosomal peptides was ~ 70% lower than for facultatively anaerobic or aerobic bacteria, suggesting a relatively lower secondary metabolite potential of obligate anaerobes. Nevertheless, 25% of obligate anaerobes were capable of synthesizing RiPPs, and 33% were capable of synthesizing polyketides and non-ribosomal peptides (Letzel et al. 2013, 2014). As previously noted, these in silico genome mining tools rely on sequence similarity to known BGCs, which largely originate from aerobes. Tentatively, in silic*o* studies under-represent the actual secondary metabolite biosynthetic capability of obligate anaerobes. Overall, obligate anaerobes, including those from the gut, remain a novel source for secondary metabolites, and there is more to discover about natural product chemistry from the anaerobic world.

Previous reviews on gut microbial secondary metabolites predominantly discuss gut bacteria. However, recent evidence suggests gut fungi also possess secondary metabolite potential. For example, *Basidiobolus* from amphibian guts, produces secondary metabolites likely acquired by horizontal gene transfer from bacteria (Tabima et al. 2020). Additionally, two novel, antifungal glycosides fusintespyrone A (Fig. 1) and cerevisterolside A were discovered from the human intestinal fungus *Fusarium* sp. (Zhang et al. 2024b). While *Basidiobolus* and *Fusarium* are facultative gut anaerobes, anaerobic gut fungi (phylum *Neocallimastigomycota*) are obligate anaerobes from the guts of some large herbivores, such as ruminants. Through in silico genome mining and multi-omics data analysis, Swift et al. showed that anaerobic gut fungi possess untapped secondary metabolite potential (Swift et al. 2021b). Additional co-cultivation studies of anaerobic gut fungi with rumen bacteria or methanogens showed differential regulation of predicted secondary metabolite genes, suggesting possible secondary metabolite roles in community membership (Swift et al. 2019, 2021a). Overall, these recent studies show an increasing awareness of gut fungi as a source of novel secondary metabolites.

### Anaerobic biofoundries enable high-throughput methods for studying secondary metabolites from anaerobic gut microbes

**Fig. 4:**
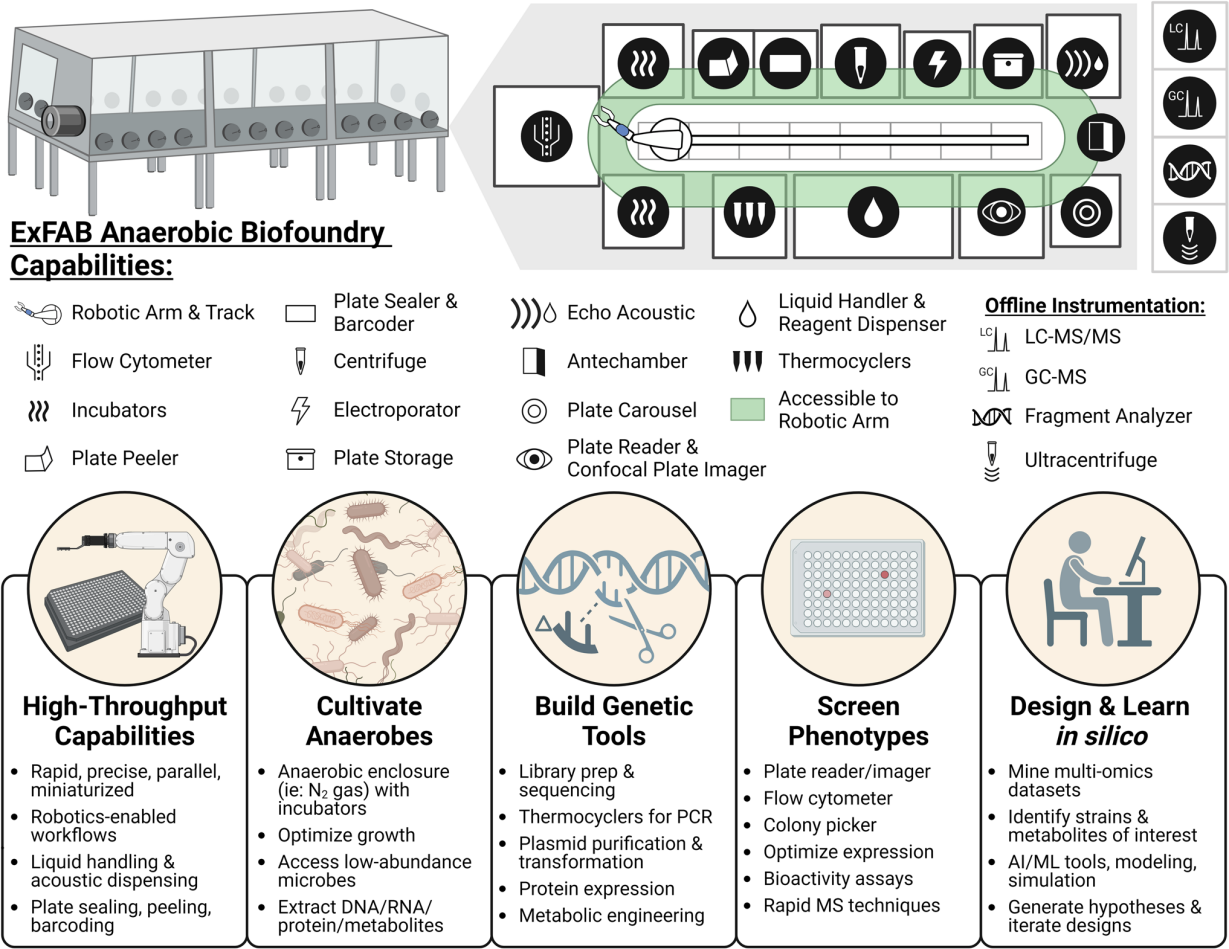
Biofoundries like ExFAB follow the Design-Build-Test-Learn paradigm to rapidly engineer, evaluate, and optimize biological systems through continuous, data-driven refinement. These facilities often include robotic sample management, liquid handlers, analytical instrumentation, and software systems to orchestrate complex workflows involving multiple devices operating in unison. Biofoundries are ideal for clearly defined, repetitive tasks, rather than exploratory work that requires flexibility. Figure was created with BioRender.com

Laboratory automation is well-established for accelerating synthetic biology research. Laboratory automation focuses on linking multiple automated unit operations into complete experimental workflows, with the goal of fully autonomous operation (Gurdo et al. 2023). A key example of laboratory automation is the biofoundry—an automated facility focused on enabling synthetic biology (Fig. 4) (Hillson et al. 2019). Approximately 40 publicly-accessible biofoundries are in operation globally, many of which participate in the Global Biofoundry Alliance to promote interlaboratory collaboration (Hillson et al. 2019). However, the majority of these facilities have limited capability for anaerobic experimentation. Two recent biofoundries in the United States are specifically designed to support complete workflows under anaerobic conditions: the NSF BioFoundry for Extreme & Exceptional Fungi, Archaea, and Bacteria (ExFAB) and LanzaTech (exfab.org, lanzatech.com). Successfully automated or semi-automated systems require minimal human input, allowing for their continuous operation in sealed enclosures with controlled atmospheric compositions, ideal for anaerobic studies.

Anaerobic biofoundries offer clear advantages for both culture-dependent and (meta)genome-dependent workflows for secondary metabolite discovery from gut anaerobes (Fig. 2). For culture-dependent workflows, automated sampling and liquid handling enable high-throughput testing to optimize microbial cultivation and secondary metabolite production, while reducing consumable costs by miniaturizing experiments to 96-, 384-, and 1536-well plate formats (Singleton et al. 2019; Otero-Muras and Carbonell 2021; Huang et al. 2023). Automation also enhances OSMAC and other elicitor-based strategies to induce silent BGCs, by allowing rapid testing of more challenge compounds (Xu et al. 2019). Subsequently, high-throughput metabolomic and bioactivity screens identify secondary metabolites of interest and guide downstream optimization efforts (Zoffmann et al. 2019; Kontou et al. 2023; Liu et al. 2024). Historically slow and labor-intensive, comprehensive chemical analysis of cell culture material is now expedited by rapid mass spectrometry (MS) techniques, including acoustic ejection MS (Zhang et al. 2021) and desorption MS technologies (Blincoe et al. 2020; Morato et al. 2021; Dueñas et al. 2023), with cycle times on the order of seconds. These techniques require minimal sample preparation and are readily integrated into biofoundry workflows with automated liquid handling. For (meta)genome-based workflows, biofoundries streamline molecular cloning protocols for rapid generation of DNA constructs (Rosch et al. 2024), enabling efficient capture of target BGCs from (meta)genome databases. Large construct libraries with novel BGCs can be inserted into panels of candidate host strains via automated transformation techniques, such as microwell plate electroporation (Rosch et al. 2024) and precise CRISPR/Cas-based genome editing (Tong et al. 2021), enabling efficient expression of oxygen-sensitive biosynthetic machinery in anaerobic heterologous systems.

Anaerobic biofoundries are a recent development that hold promise in targeting the untapped potential of gut anaerobes. However, designing and operating an anaerobic biofoundry presents several challenges. Biofoundries require significant upfront cost and effort to build, train personnel, and develop workflows (Holowko et al. 2021). Environmental chambers to house automated instrumentation are often custom-designed, larger, and more complex than widely available chambers, making initial implementation and long-term maintenance costly. Instrumentation is not routinely tested for compatibility with an anaerobic atmosphere (low humidity, high N_2_, etc.) and may suffer from inconsistent function or reduced lifetime under these unique conditions. Of particular concern is the potential for hydrogen sulfide production by sulfate-reducing microbial strains, which can accumulate in a closed chamber and quickly degrade sensitive electronic components if active gas purification is not employed (Jung et al. 2022). Maintaining system sterility presents an additional challenge, especially when spore-forming bacterial or fungal strains are introduced, as access for manual disinfection is often limited. Rigorous procedures involving multiple disinfection methods (surface disinfection, UV irradiation, hydrogen peroxide or ozone gas exposure, etc.) are often required to minimize microbial contamination (Epelle et al. 2023). Despite these challenges, successful implementation of anaerobic biofoundry workflows promises to revolutionize studies on gut microbiomes and their metabolism.

## Conclusion

Gut microbes are a largely untapped source of secondary metabolites that impact host health and present therapeutic and biotechnological applications. Among these microbes, gut fungi and obligate anaerobes are particularly under-explored yet promising for novel metabolites. Recent advances in anaerobic biofoundries offer opportunities to overcome experimental bottlenecks in working with gut anaerobes. Such automated workflows hold promise to advance our understanding of gut anaerobe cultivability, expand the knowledge base of BGCs, reveal metabolic strategies of gut anaerobes, implement high-throughput heterologous systems for oxygen-sensitive biosynthetic machinery, and accelerate phenotypic screens for bioactive metabolites of interest. Overall, the latest innovations for secondary metabolite discovery from the anaerobic gut microbiome will continue to reveal the unknown natural product chemistry of the anaerobic world.
